# Anti-Diabetic and Antioxidant Activities of Red Wine Concentrate Enriched with Polyphenol Compounds under Experimental Diabetes in Rats

**DOI:** 10.3390/antiox10091399

**Published:** 2021-08-31

**Authors:** Mariya Sabadashka, Dariya Hertsyk, Paulina Strugała-Danak, Anita Dudek, Olena Kanyuka, Alicja Z. Kucharska, Leonid Kaprelyants, Nataliia Sybirna

**Affiliations:** 1 Department of Biochemistry, Ivan Franko National University of Lviv, 1, Universytetska St., 79000 Lviv, Ukraine; Dariya.Hertsyk@lnu.edu.ua (D.H.); olena.kaniuka@lnu.edu.ua (O.K.); nataliya.sybirna@lnu.edu.ua (N.S.); 2 Department of Physics and Biophysics, Wrocław University of Environmental and Life Sciences, C. K. Norwida 25, 50-375 Wrocław, Poland; 113767@student.upwr.edu.pl; 3 Department of Fruit, Vegetable and Plant Nutraceutical Technology, Wrocław University of Environmental and Life Sciences, Chełmońskiego St. 37, 51-630 Wrocław, Poland; alicja.kucharska@upwr.edu.pl; 4 Department of Biochemistry, Microbiology and Nutrition Physiology, Odessa National Academy of Food Technologies, Kanatnaya St., 65039 Odessa, Ukraine; leonid@onaft.edu.ua

**Keywords:** red wine concentrate, polyphenolic compounds, experimental diabetes mellitus, antidiabetic, antioxidant, liposomes

## Abstract

We obtained red wine concentrate, which was enriched with natural polyphenolic compounds (PC concentrate). The main purpose was to study the hypoglycemic and antioxidant effects of the red wine concentrate, and its impact on key hematological parameters of rats with experimental diabetes mellitus. While administrating the red wine concentrate to rats with diabetes, partial recovering of glucose tolerance was promoted, as well as normalization of glycated hemoglobin level, an increase in the quantity of erythrocytes and hemoglobin concentration. PC concentrate had anti-radical effect, which was determined using 2,2-diphenyl-1-picrylhydrazylradical (DPPH) method and effectively inhibited oxidation of phosphatidylcholine liposomes, induced by 2,2′-azobis(2-amidinopropane) dihydrochloride (AAPH) as a free radical generator. It was also confirmed that PC concentrate had antioxidant properties in vivo. The contents of lipid peroxidation and protein oxidation products, the activity of catalase, and glutathione peroxidase (GPx) were increased in the plasma of rats with diabetes mellitus. At the same time, the activity of superoxide dismutase (SOD) was decreased. The concentrate of red wine had a corrective effect on investigated indicators and caused their normalization in plasma of diabetic animals.

## 1. Introduction

Scientists from all over the world are searching for different compounds with potential anti-diabetic and antioxidant activities. Compounds with such properties can become the basis for different drugs to treat and prevent the development of various diseases. Studies that have shown unwanted or adverse effects of synthetic antioxidants have forced scientists to focus their researches on exploring the natural sources with reasonable antioxidant potential [1]. There is a variety of such compounds, but polyphenols are the most perspective.

Polyphenolic compounds are plant origin secondary metabolites and are contained in fruits, vegetables, cereals, and coffee, etc. The researchers have verified the role of polyphenols in the remedy of cardiovascular disease, osteoporosis, neurodegenerative disease, cancer, and diabetes mellitus [2].

Red wine is a rich source of polyphenolic compounds [3]. Almost 63% of all phenolic substances from grape seeds and skins are converted into wine during its production. So wine can be considered as one of the most effective natural remedies provided the consumption of an optimal dose [4,5]. Antioxidant, vasodilation, anti-oncological, anti-inflammatory, anti-allergic, antiviral, and estrogenic effects of polyphenols are shown. Polyphenols inhibit phospholipase A2, cyclooxygenase, lipoxygenase, glutathione reductase, and xanthine oxidase. Polyphenols also can chelate metal ions [6]. In vitro wine polyphenols scavenge free radicals, in particular superoxide anion radical and hydroxyl radical, and inhibit lipid peroxidation reactions [7]. All the above justifies the possibility of using phenolic compounds as a basis for creating new anti-diabetic drugs.

Diabetes mellitus is a chronic disease that requires appropriate medical care and constant training of patients to control the quality of food and a moderately active lifestyle. According to WHO statistics, there will be about 500 million people on the planet suffering from diabetes by 2025. It is known that patients with diabetes spend about three times more money on maintaining their health than a person without diabetes [8]. Diabetes mellitus is accompanied by complications associated with dysfunctions of the limbs, retina, kidneys, nerves, heart, and blood vessels. Complications of diabetes not only worsen the quality of life of patients but also cause death. The main reasons for the progress of complications are a chronic inflammatory process [9,10], lesions of the oxygen-transport system and hypoxia [11,12], as well as redox imbalance [13,14,15].

Considering the etiology and pathogenesis of diabetes, researchers have investigated the possibility of using polyphenols from grapes and grape wine as compounds with potential anti-diabetic activity [16,17,18,19]. It is important to note, that the anti-diabetic effect of natural polyphenols is one of the most intensely studied [20].

At the same time, the mechanisms of action of the polyphenolic complex of red grape wine on key hematological indexes are not fully understood. Therefore, the aim of the study was to investigate the biochemical mechanisms of anti-diabetic and antioxidant effect of natural polyphenol complex concentrate of red grape wine (PC concentrate) from Ukrainian vineyard in experimental diabetes mellitus.

## 2. Materials and Methods

### 2.1. Chemicals and Standards

Streptozotocin, gallic acid, 2,2-diphenyl-1-picryl hydrazyl radical (DPPH), 6-hydroxy-2,5,7,8-tetramethyl chroman-2-carboxylic acid (Trolox), quercetin, phenazine metasulfate, NADH, tert-butyl hydroperoxide, acetonitrile, methanol, acetic acid, 3-[p-(6-phenyl)-1,3,5-hexatrienyl] propionic acid (DPH-PA), 2,2′-azobis(2-amidinopropane) dihydrochloride (AAPH) and L-α-phosphatidylcholine (from egg yolk) were purchased from Sigma-Aldrich (Steinheim, Germany). Acetonitrile for liquid chromatography-mass spectrometry was purchased from POCh (Gliwice, Poland). Cyanidin 3-*O*-glucoside (Cy glc), caffeic acid (CA), gallic acid (GA), protocatechuic acid (PA) were purchased from Extrasynthese (Lyon Nord, France). Trans-caftaric acid was purchased from Cayman Chemical Company (Michigan, EUA, Ann Arbor, MI, USA). A commercially available kit for glucose concentration measurement was purchased from Filisit diagnostics (Kyiv, Ukraine). Other chemical reagents were produced in Ukraine. All reagents were of analytical grade.

### 2.2. Wine Material Used in Research

Dry red wine with a volume fraction of alcohol of 14% was used in the study. The wine was received from Odesa Black grapes harvested at the research and production enterprise LLC “NIVA” (Odesa region, Ovidiopol district; GPS coordinates: 46°22′35.4″ N, 30°36′58.3″ E) in 2017. Table 1 shows the physical and chemical properties of dry wine used in the experiment. Procedures were conducted according to “Wines. General specifications. SCSU 4806:2007” [21].

### 2.3. Preparation of Concentrate of Red Wine Enriched with Natural Polyphenolic Compounds

Vacuum evaporation of wine was performed on a rotary evaporator Laborota 4001 (Schwabach, Germany) at a temperature of 40 °C to obtain PC concentrate. Evaporation was performed from 100 mL of red grape wine to 30 mL of concentrate [22]. Then ethanol was added to a final concentration of 30% to improve the solubility of polyphenols in PC concentrate. Stabilization of PC concentrate was also performed by adding the biogenic surfactants, the products of *Pseudomonas* sp. PS-17 biosynthesis (PS complex). PS biocomplex was added to a final concentration of 3%.

### 2.4. Identification and Quantification of Compounds of PC Concentrate by the UPLC-qTOF-MS/MS and HPLC-PDA Methods

The UPLC-qTOF-MS/MS method was previously described by Kucharska et al. [23]. Identification of compounds was performed using the Acquity ultra-performance liquid chromatography (UPLC) system, coupled with a quadrupole-time of flight (Q-TOF) MS instrument (UPLC/Synapt Q-TOF MS, Waters Corp., Milford, MA, USA), with an electrospray ionization (ESI) source. The separation was achieved on an Acquity BEH C18 column (0.1 m × 0.0021 m i.d., 0.0000017 m; Waters). Anthocyanins were explored in the positive mode while phenolic acids–in the negative mode before and after fragmentation.

The HPLC-PDA method was previously described by Kucharska et al. [23]. The quantification analysis was performed using a Dionex (Germering, Germany) system, equipped with the diode array detector model Ultimate 3000, quaternary pump LPG-3400A, autosampler EWPS-3000SI, thermostated column compartment TCC-3000SD, and controlled by Chromeleon v.7.2.9 software (Thermo Scientific Dionex, Sunnyvale, CA, USA). The Cadenza Imtakt column CD-C18 (0.075 × 0.0046 m, 0.000005 m) was used. Anthocyanins were detected at 520 nm, caftaric and coutaric acid at 320 nm, gallic acid at 280 nm, and protocatechuic acid at 254 nm. Anthocyanins were expressed as cyanidin 3-O-glucoside, hydroxycinnamic acids esters as caffeic acid, and gallic acid and protocatechuic acid as standards. The results were expressed as mg per 100 mL [24,25,26,27].

### 2.5. DPPH Radical Scavenging Assay

The antioxidative capacity of PC concentrate was determined by the percentage of reduction of 2, 2-diphenyl-1-picrylhydrazyl radical (DPPH) [28]. Working solution of 0.1 mM DPPH in methanol was prepared immediately before using.

6-hydroxy-2,5,7,8-tetramethyl chroman-2-carboxylic acid (trolox) and quercetin were used as standard samples. We used the following concentrations of standards (μg/mL): 0, 12.5, 25, 50, 75, 100, 125, 150, 175, 200.

We prepared a series of dilutions of PC concentrate containing the following concentrations of polyphenolic compounds (μg/mL): 0, 12.5, 25, 50, 75, 100, 125, 150, 175, 200.

1.45 mL of DPPH solution was added to 0.05 mL of each sample. After 30 min of incubation in the dark at room temperature, extinction was measured at a wavelength of 517 nm. The percentage of reduction of DPPH was calculated using the formula:

$$\%\;DPPH\;reduction=\frac{\left(A_{0}-A_{1}\right)}{A_{0}}\times 100$$

where *A*_0_ is the value of light absorption of DPPH solution without antioxidants (control), *A*_1_ is the value of light absorption of DPPH solution containing the antioxidants (experiment).

The concentration of PC concentrate providing 50% reduction of DPPH (EC_50_) was calculated from the graph plotting the percentage of reduced DPPH against the sample concentration.

### 2.6. Liposome Oxidation Assay

The antioxidant activity of PC concentrate towards phosphatidylcholine liposomes was determined with the fluorimetric method according procedure described earlier by Strugała et al. [29] with minor modifications.

The lipids were dissolved in chloroform (100 mg/mL), evaporated to dryness under nitrogen and under vacuum for another 60 min. Subsequently, a phosphate buffer of pH 7.4 was added and liposomes were formed by mechanical shaking. Then SUVs were formed using a 20 kHz sonicator for 15 min. The vesicle suspension obtained was used as the source of liposomal membranes as final concentration equal 0.1 mg/mL.

For study of antioxidant activity, the relationship between DPH-PA probe fluorescence intensity and concentration of free radicals was used (supplied by AAPH which, under the influence of 37 °C, decomposed into alkyl radicals). Free radicals released in the process of liposome oxidation caused quenching of DPH-PA probe fluorescence, decreasing the fluorescence intensity. As a measure of the extent of lipid oxidation we used relative fluorescence, i.e., the ratio of oxidized probe fluorescence to the initial fluorescence of the probe. The PC concentrate scavenged free radicals and thus caused a lower rate of DPH-PA fluorescence decrease. As a control we used the relative fluorescence of liposomes suspension that contained the DPH-PA probe, oxidized by AAPH radical, and the blank sample was the relative fluorescence of a suspension of the same concentration but not oxidized by AAPH.

Liposomes in phosphate buffer (pH 7.4), incubated for 30 min in the dark with the addition of DPH-PA probe at a concentration of 1 µM. Oxidation was initiated just before the measurement with AAPH at a concentration of 1 M and in the presence of different concentrations of antioxidant (32–158 µg/mL). The wavelengths of excitation and emission of the probe were, respectively, λex = 365 nm, λem = 430 nm. The percentage inhibition of lipid oxidation was calculated on the basis of the following formula:

$$\%\;of\;inhibition=\frac{\left(F_{S}-F_{C}\right)}{\left(F_{B}-F_{C}\right)}\times 100\%$$

where *F_S_* refers to relative fluorescence of the probe oxidized by AAPH in the presence of antioxidant; *F_C_* refers to relative fluorescence of control sample oxidized by AAPH without antioxidant; *F_B_* refers to relative fluorescence of the blank sample.

All measurements were performed for four independent preparations (n = 4) using a fluorimeter (Cary Eclipse, Varian, San Diego, CA, USA).

### 2.7. Animals

The experiments were conducted on Wistar white rats 150–180 g in weight. All the procedures with the animals were conducted in accordance with General Principles of Animal Treatment, approved by the First National Congress on Bioethics (Kyiv, Ukraine, 2001),which are agreed with the guidelines of Directive 2010/63/EU of the European Parliament on the protection of animals used for scientific purposes and the Law of Ukraine “On Protection of Animals from Cruelty” of 26 February 2006, and approved by the Ethics Committee of Ivan Franko National University of Lviv, Ukraine(Certificate No 22-07-2021 of 21 July 2021). The animals were kept in the vivarium and had free access to food and water.

Rats were randomly divided into four groups: 1–normal untreated control animals (hereinafter called C); 2–animals that were per os administrated PC concentrate (hereinafter called C + PC); 3–rats with experimental diabetes mellitus (hereinafter called DM), 4–animals with experimental diabetes mellitus that were per os administrated PC concentrate (hereinafter called DM + PC). Each group included 6–8 rats.

An aqueous solution of PC concentrate (final volume 1 mL) was administered per os using a tube daily for 14 days. The total dose was 45 mg of polyphenolic complex per 1 kg of body weight of animals of the 2nd and 4th experimental groups. The dose corresponds to the theoretical average concentration of polyphenols contained in 300 mL of red wine (which is considered as the daily norm for a person weighing 70 kg).

### 2.8. Induction of Diabetes Mellitus Type 1

Following 18 h of starvation, experimental diabetes mellitus was induced by intraperitoneal administration of streptozotocin dissolved in 10 mM citrate buffer (pH 5.5) at a dose of 0.055 g/kg of body weight. The development of diabetes was monitored by blood glycemia level. Blood glycemia was determined after 72 h of streptozotocin injection. Animals with a glucose level higher than 12 mM were used in the further experiment. We started to administrate the PC concentrate on the 14th day after induction of diabetes.

### 2.9. Determination of Glucose Concentration

Determination of fasting glucose was performed using a commercially available kit (Filisit diagnostics, Kyiv, Ukraine). The blood was collected from the tail vein of rats after 18 h of starvation for each experimental group.

### 2.10. The Glucose Tolerance Test

The oral glucose tolerance test (OGTT) was performed after 18 h of starvation. Fasting glucose (0 min) blood samples were collected from the tail vein of rats after 18 h of starvation. Glucose loading was performed by oral administration of glucose solution at the rate of 1 g of glucose per 1 kg of body weight to animals of each experimental group. Blood samples were then taken at 15, 30, 45 and 60 min after glucose administration.

Index of the area under the glycemic curve (AUCglu) was calculated. The index shows a general increase in glucose concentrations after the glucose load. The area under the glycemic curve was calculated by the trapezoid rule [30].

### 2.11. Blood Collection

The rats from all experimental groups were entered the surgical stage by ether anesthesia on the 29th day of the experiment. Samples collection was carried out after the decapitation of the animals. The blood was collected into a porcelain cup. Heparin was used as an anticoagulant (a final dilution of heparin: whole blood = 1:100).

### 2.12. Determination of the Number of Erythrocytes

The number of erythrocytes was determined according to the method of counting in hemocytometer using a MICROmed(Poltava, Ukraine) light microscope [31,32,33].

### 2.13. Determination of Total Hemoglobin Concentration

For analysis, 0.02 mL of blood was mixed with 5 mL of transforming solution (containing 1 g NaHCO_3_; 0.2 g K_3_[Fe(CN)]_6_ and 0.5 mL acetone cyanohydrin in 1.0 L of solution). After 20 min of incubation, a light absorption was measured at 540 nm against water on a spectrophotometer (Helios Epsilon, Waltham, MA, USA). The hemoglobin concentration was calculated by the formula:

$$C_{Hb}=E_{540}\times 36.77\;(g\%)$$

where *C_Hb_* means hemoglobin concentration; *E*_540_ means light absorption of the blank sample; 36.77–recalculation coefficient [32,33].

### 2.14. Isolation of Erythrocytes

Erythrocytes were separated from blood by centrifugation at 3000 rpm for 10 min. Erythrocytes were washed with chilled 0.85% NaCl at 3000 rpm for 5 min three times to avoid cells lysis [32].

### 2.15. Obtaining of Erythrocytes Lysate

To prepare the hemolysates, distilled water was added to 1 mL of erythrocytes suspension in a ratio of 1:2 and stirred for a few mins at room temperature. The obtained hemolysates were used to determine the content of glycated hemoglobin.

### 2.16. Determination of Glycated Hemoglobin Content

The content of glycated hemoglobin (HbA1c) in erythrocytes was determined after adding 1 mL of 0.3 M oxaloacetic acid to 2 mL of hemolysate. The mixture was incubated at 95 °C for 1 h. After cooling the mixture, 1 mL of 40% trichloroacetic acid (TCA) was added, shaken, and centrifuged for 10 min at 3000 rpm. To 2 mL of the supernatant was added 0.5 mL of 0.05 M thiobarbituric acid (TBA). Obtained mixture was incubated at 40 °C for 40 min. After that, the optical density of the test sample was measured at 443 nm. The optical density value of 0.029 corresponds to 1% HbA1c in a sample [32,33].

### 2.17. Determination of Protein Concentration by Lowry Method

Protein concentration was determined by the conventional Lowry method [34]. Reagent “A” (containing 20 g of Na_2_CO_3_ dissolved in 1 L of 0.1 M NaOH) and reagent “B” (containing 10 g of sodium citrate and 5 g of CuSO_4_ × 5H_2_O dissolved in 1 L of H_2_O) were prepared. Reagent “C” was prepared directly before using by mixing 50 mL of reagent “A” and 1 mL of reagent “B”.

To 0.4 mL of the sample, 2 mL of reagent “C” was added. The mixture was stirred and after 10 min 0.2 mL of Folin–Ciocalteu reagent was added. After 30 min of incubation in the dark at room temperature, extinction was measured at a wavelength of 750 nm. The protein concentration was determined using the calibration curve. The results were expressed in mg per mL.

### 2.18. Methods of Studyingthe Activity of Antioxidant Enzymes

#### 2.18.1. Determination of Superoxide Dismutase Activity

To 0.1 mL of plasma were added 0.9 mL of distilled water, 0.5 mL of absolute alcohol, 0.25 mL of chloroform, and 300 mg of KH_2_PO_4_. After that, the mixture was shaken for 5 min and centrifuged at 2500× *g* for 30 min.

The incubation solution (39 mM EDTA-Na, 114 mM nitrotetrazolium blue (meta form), 54 mM phenazine metasulfate in 0.15 M phosphate buffer, pH = 7.8) containing 8 mM NADH, was added to the samples. A lysis buffer was added to the blank instead of the supernatant. The reaction was carried out at a temperature of 24–25 °C for 10 min. The extinction was measured at 540 nm [35].

The obtained values were calculated using the calibration curve into International Units of Activity (U). SOD activity was expressed in International Units of Activity per 1 mg of protein.

#### 2.18.2. Determination of Catalase Activity

Catalase activity was determined after adding 2 mL of 0.03% H_2_O_2_ to 100 μL of plasma. After incubation at 37 °C for 10 min, the reaction was stopped by 1 mL of 0.125 M H_2_SO_4_. After that 1 mL of 4% (NH_4_)_2_MoO_4_ was added to samples. Samples were centrifuged at 10,000 rpm for 10 min. The color intensity of the samples was determined spectrophotometrically at 410 nm [36].

#### 2.18.3. Determination of Glutathione Peroxidase Activity

To determine the activity of glutathione peroxidase (GPx), 100 μL of plasma were incubated at 37 °C for 10 min with 830 μL of 0.1 M Tris-HCl buffer (pH 8.5), which contained 6 mM EDTA, 12 mM sodium azide (NaN_3_) and 4.8 mM reduced glutathione (GSH). Then, 70 μL of 20 mM tert-butyl hydroperoxide were added to the samples and incubated for 5 min. The reaction was stopped by adding 20% TCA. The samples were centrifuged at 10,000 rpm for 10 min. To 20 μL of the supernatant were added a similar volume of Elman’s reagent (0.01 M 5,5′-dithiobis-2-nitrobenzoic acid diluted in methanol) and 2 mL of 0.1 M Tris-HCl buffer (pH 8.5). The mixture was incubated for 5 min, after that the optical density of samples was measured at 412 nm. The results were expressed in nmol GSH for1 min per 1 mg of protein [37].

### 2.19. Determination of the Content of the Products of Oxidative Modification of Proteins

To determine the level of oxidative modified proteins to 0.2 mL of plasma were added 0.8 mL of 0.85% NaCl, 1 mL of 0.1 M 2,4-dinitrophenylhydrazine dissolved in 2 M HCl, and 1 mL of 10% TCA. The samples were incubated for 1 h at 37 °C with further centrifugation for 10 min at 3000 rpm. The precipitate was washed three times with 5% TCA. Afterward, the precipitate was incubated for 5 min with 5 mL of 8 M urea in a boiling water bath until complete dissolution. The optical density of the formed dinitrophenylhydrazones complexes was measured at 370 nm (to determine the level of products of neutral character) and 430 nm (to determine the level of products of basic character). The content of phenylhydrazones of neutral character (370 nm) was calculated using the molar extinction coefficient ε = 220 m^−1^ × M^−1^ and expressed in μmol per g of protein. The content of phenylhydrazones of basic character (430 nm) was expressed in conventional units (c. u.) per 1 g of protein [38].

### 2.20. Determination of the Content of Lipids Peroxidation Products

The level of lipid peroxidation was assessed by determining the content of acid reactive substances (TBARS). To 0.2 mL of plasma were added 3 mL of 10 mM K, Na-phosphate buffer (in 125 mM KCl, pH = 7.4) and 0.5 mL of 1 mM KMnO_4_. To induce lipid peroxidation 0.5 mL of 10 mM FeSO_4_ were added twice with an interval of 10 min. The reaction was stopped with 1 mL of 20% TCA and samples were centrifuged. To 1 mL of supernatant were added 0.25 mL of 1 M HCl and 0.5 mL of 0.7 mM TBA and incubated at 100 °C for 20 min. Then the sample was cooled and the light absorption was measured at 532 nm [39]. The results were expressed in nmol per 1 mL of plasma and recalculated in % (where the control value was 100%).

### 2.21. Statistical Analysis of Results

Statistical analysis of the results was carried out using Origin Pro. The calculation of basic statistical parameters was performed by direct quantitative data obtained from the study (arithmetic mean–AM, the standard deviation of the arithmetic mean–SD).

To assess the reliability of the difference between statistical characteristics of the two alternative data sets, we performed Student’s *t*-test. The difference was considered significant different under *p* < 0.05.

## 3. Results

### 3.1. Qualitative and Quantitative Analysis of Red Wine Polyphenol Complex Concentrate

The results of qualitative and quantitative identification of the phenolic compounds of wine and PC concentrate are summarized in Table 2. The compounds were tentatively identified considering their retention times, elution order, spectra of the individual peaks (UV/Vis, MS, MS/MS), and data from the literature [40,41]. In our research, we determined 20 anthocyanins and 4 phenolic acids. Anthocyanins and other phenolic compounds were detected using the positive and negative modes, respectively. Five monoglucosides of delphinidin, cyanidin, petunidin, peonidin, and malvidin, five acetyl derivatives of delphinidin, cyanidin, petunidin, peonidin, and malvidin, four *p*-coumaroyl derivatives of cyanidin, petunidin, peonidin, and malvidin, five Vitisin A-type derivatives, and one glucoside malvidin adduct with 4-vinyl phenol were identified among the anthocyanins.

Monoglucosides (compounds 1–5) were identified in analyzed samples by the presence of the pseudmolecular ions [M + H]^+^ at *m/z* 465.1026, 449.1092, 479.1210, 463.1239, and 493.1339, respectively and the fragments [M + H–162]^+^. The loss of a glucose unit (162 Da) generated the aglycone ions that corresponded to the molecular ions of the delphinidin, cyanidin, petunidin, peonidin, malvidin moieties, respectively.

In investigated samples were identified acetylated anthocyanins. The compounds 7, 10–13 exhibited a fragment ions [M + H–162–42]^+^ at *m/z* 507.1139, 491.1145, 521.1305, 505.1350, 535.1441, respectively, after loss of an acetyl-glucoside, while compounds 16–19 corresponded to the loss of a *p*-coumaryl-glucoside unit (308 Da) after fragments. The compounds 7, 10–13, 16–19 were identified as: delphinidin 3-*O*-(6-*O*-acetyl)-glucoside, cyanidin 3-*O*-(6-*O*-acetyl)-glucoside, petunidin 3-*O*-(6-*O*-acetyl)-glucoside, peonidin 3-*O*-(6-*O*-acetyl)-glucoside, malvidin 3-*O*-(6-*O*-acetyl)-glucoside, cyanidin 3-*O*-(6-*O*-*p*-coumaroyl)-glucoside, petunidin 3-*O*-(6-*O*-*p*-coumaroyl)-glucoside, peonidin 3-*O*-(6-*O*-*p*-coumaroyl)-glucoside, and malvidin 3-*O*-(6-*O*-*p*-coumaroyl)-glucoside, respectively. For compound 20, the pseudomolecular ion [M + H]^+^ and fragment ion [M + H–162]^+^ were 609.1618 and 447.1086, respectively. Thus, it was tentatively identified as malvidin 3-*O*-glucoside adduct with 4-vinyl phenol.

In samples, we identified vitisin A-type derivatives i.e., pyruvic acid with peonidin 3-*O*-glucoside, malvidin 3-*O*-glucoside, malvidin 3-*O*-(6-*O*-acetyl)-glucoside, peonidin 3-*O*-(6-*O*-*p*-coumaroyl)-glucoside, and malvidin 3-*O*-(6-*O*-*p*-coumaroyl)-glucoside derivatives. Pyruvic acid allows the formation of large amounts of vitisin A [24]. This acid is released into the pomace by yeast during alcoholic fermentation [26,27], and as well as it can also be released by lactic bacteria during malolactic fermentation.

Among the phenolic acids there were identified hydroxybenzoic acids just like gallic acid (21) with a pseudomolecular ion at *m/z* 169.0116 and protocatechuic acid (22) with a pseudomolecular ion at *m*/*z* 153.0164 and hydroxycinnamic acids esters. Compounds 23 and 24 were identified as caftaric acid and coutaric acid with a pseudomolecular ion at *m/z* 311.0404 and 295.0457, respectively. Their fragment ions at *m/z* 179.0342 (23) and 163.0379 (24) were obtained after the loss of tartaric acid moiety (44 Da) from hydroxycinnamic ester. This is consistent with the literature data [40,42].

The amount of total phenolic compounds varied not much between wine 1 (239.35 mg/100 mL) and PC concentrate 2 (257.43 mg/100 mL) obtained from this wine (Table 2). The concentration of anthocyanins and other compounds was 97.44–104.13 mg/100 mL and 141.91–153.30 mg/100 mL, respectively. The dominant anthocyanins were malvidin 3-*O*-glucoside (39.28–42.18 mg/mL) and petunidin 3-*O*-glucoside (13.92–14.91 mg/mL). Among phenolic acids, caftaric acid was dominating (66.85–69.37 mg/100 mL) and then coutaric acid (41.97–44.12 mg/100 mL), gallic acid (29.10–34.90 mg/100 mL). Protocatechuic acid was present in samples in small amounts (3.99–4.91 mg/100 mL).

### 3.2. The Effect of Red Wine Polyphenol Complex Concentrate on Glycemia under Diabetes Mellitus

**Figure 1:**
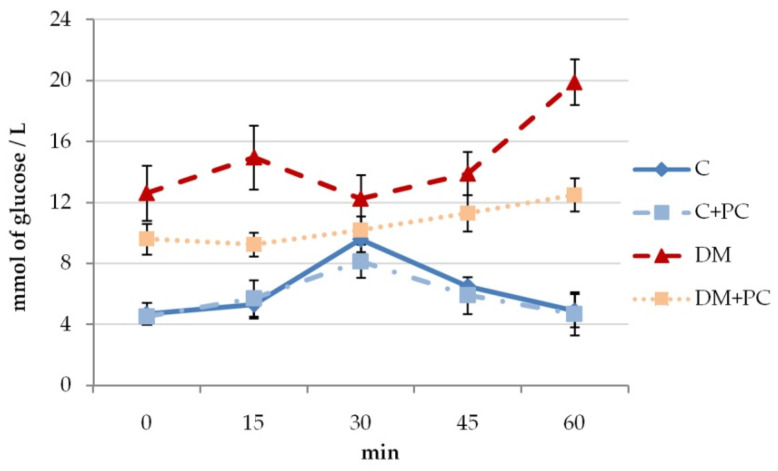
Glucose tolerance test of control and diabetic rats, with and without PC concentrate. C–normal untreated control animals; C + PC–animals that were per os administrated PC concentrate (daily dose of 45 mg/kg bw of polyphenolic complex for 14 days); DM–rats with experimental diabetes mellitus; DM + PC–animals with experimental diabetes mellitus that were per os administrated PC concentrate (daily dose of 45 mg/kg bw of polyphenolic complex for 14 days).

**Figure 2:**
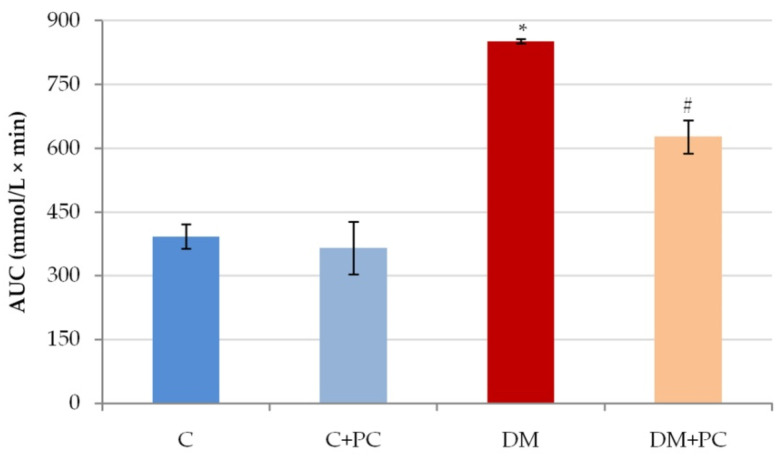
The area under the glycemic curves of control and diabetic animals, with and without PC concentrate. C–normal untreated control animals; C + PC–animals that were per os administrated PC concentrate (daily dose of 45 mg/kg bw of polyphenolic complex for 14 days); DM–rats with experimental diabetes mellitus; DM + PC–animals with experimental diabetes mellitus that were per os administrated PC concentrate (daily dose of 45 mg/kg bw of polyphenolic complex for 14 days). *-significant difference compared to control (*p* < 0.05); #-significant difference compared to diabetes mellitus (*p* < 0.05).

The greatest increase of blood glucose (by 2.2 times) in control animals was observed after 30 min after glucose consumption compared to fasting blood glucose (Figure 1). The content of glucose in the blood of control animals was normalized after 1 h after glucose loading. The same tendency was observed when analyzed glycemic curves of control animals that were treated with PC concentrate. The maximum increase in blood glucose (by 1.9 times) of animals of this experimental group was also observed at 30 min after glucose loading compared to fasting blood glucose. The index was normalized within one hour after glucose loading. However, no changes in AUCglu were detected when PC concentrate was administrated to control animals (Figure 2).

In diabetic animals (with fasting glucose of 12.6 mmol/L) blood glucose concentration increased by 1.3 times after 15 min. The maximum blood glucose concentration (by 1.7 times higher compared to fasting blood glucose) was observed after 1 h after glucose loading (Figure 1). The increase of AUCglu by 2.17 times was shown under conditions of diabetes mellitus compared to control (Figure 2).

Under PC concentrate administration to diabetic animals during 14 days fasting blood glucose was 9.6 mmol/L, then its level was characterized by an upward trend and reached a maximum after 1 h (12.5 mmol/L). Although the level of blood glucose was not decreased to the control level (Figure 1). This is confirmed by changes of AUCglu, which decreased by 1.36 times in the case of PC administration compared to untreated diabetic animals (Figure 2).

**Figure 3:**
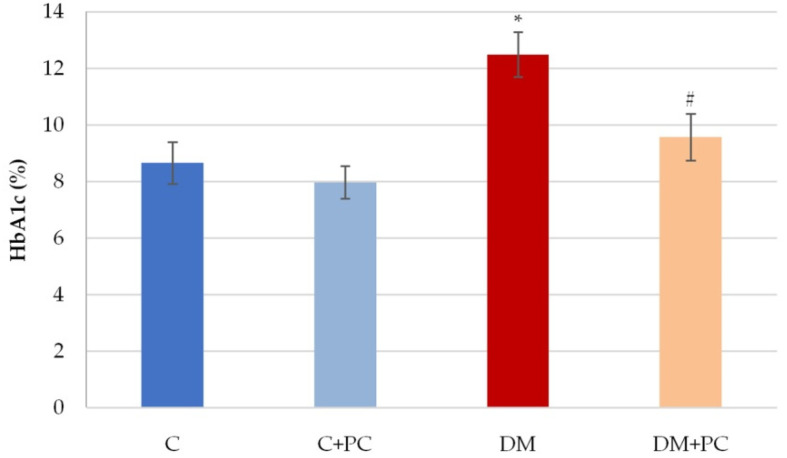
The content of glycated hemoglobin in peripheral blood of control rats, rats with experimental diabetes mellitus, with and without PC concentrate. C–normal untreated control animals; C + PC–animals that were per os administrated PC concentrate (daily dose of 45 mg/kg bw of polyphenolic complex for 14 days); DM – rats with experimental diabetes mellitus; DM + PC–animals with experimental diabetes mellitus that were per os administrated PC concentrate (daily dose of 45 mg/kg bw of polyphenolic complex for 14 days). *-significant difference compared to control (*p* < 0.05); #-significant difference compared to diabetes mellitus (*p* < 0.05).

The level of key diagnostic indicator glycated hemoglobin (HbA1c) was also studied. The increase of the level of glycated hemoglobin in peripheral blood of rats with diabetes mellitus by 44% was shown compared to control. In the case of PC concentrate consumption the level of glycated hemoglobin normalized in animals with diabetes mellitus (Figure 3).

### 3.3. Influence of Red Wine Polyphenol Complex Concentrate on Hematological Indices of Rats with Diabetes Mellitus

**Figure 4:**
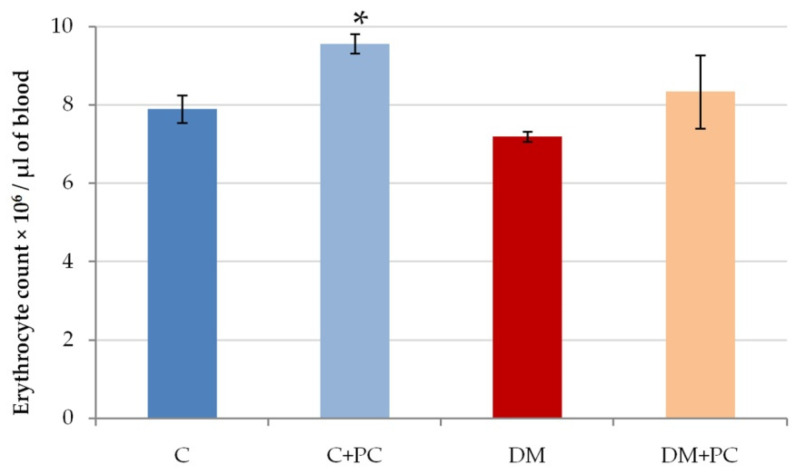
The number of erythrocytes in the blood of control rats, rats with experimental diabetes mellitus, with and without PC concentrate. C–normal untreated control animals; C + PC–animals that were per os administrated PC concentrate (daily dose of 45 mg/kg bw of polyphenolic complex for 14 days); DM–rats with experimental diabetes mellitus; DM + PC–animals with experimental diabetes mellitus that were per os administrated PC concentrate (daily dose of 45 mg/kg bw of polyphenolic complex for 14 days). *-significant difference compared to control (*p* < 0.05).

According to the obtained results (Figure 4), the number of erythrocytes is characterized by the tendency to decrease under the conditions of diabetes mellitus compared to control. Administration of PC concentrate caused an increase of the count of erythrocytes in control animals in a 1.22-fold compared to control and in diabetic animals in 1.35-fold compared to diabetes (Figure 4).

**Figure 5:**
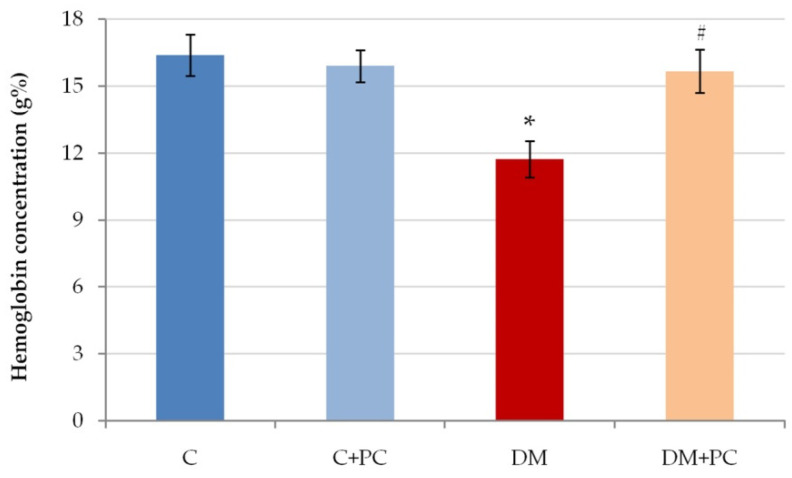
The concentration of hemoglobin in the blood of control rats, rats with experimental diabetes mellitus, with and without PC concentrate. C–normal untreated control animals; C + PC–animals that were per os administrated PC concentrate (daily dose of 45 mg/kg bw of polyphenolic complex for 14 days); DM–rats with experimental diabetes mellitus; DM + PC–animals with experimental diabetes mellitus that were per os administrated PC concentrate (daily dose of 45 mg/kg bw of polyphenolic complex for 14 days). *-significant difference compared to control (*p* < 0.05); #-significant difference compared to diabetes mellitus (*p* < 0.05).

The decrease of hemoglobin concentration by 1.4 times was shown in the blood of rats with diabetes mellitus when compared to control (Figure 5). The administration of PC concentrate normalized the hemoglobin level under diabetes mellitus conditions (Figure 5).

### 3.4. Antioxidant Effect of Red Wine Polyphenol Complex Concentrate In Vitro and In Vivo

#### 3.4.1. In Vitro Studies on the Radical Scavenging Activity of PC Concentrate

**Figure 6:**
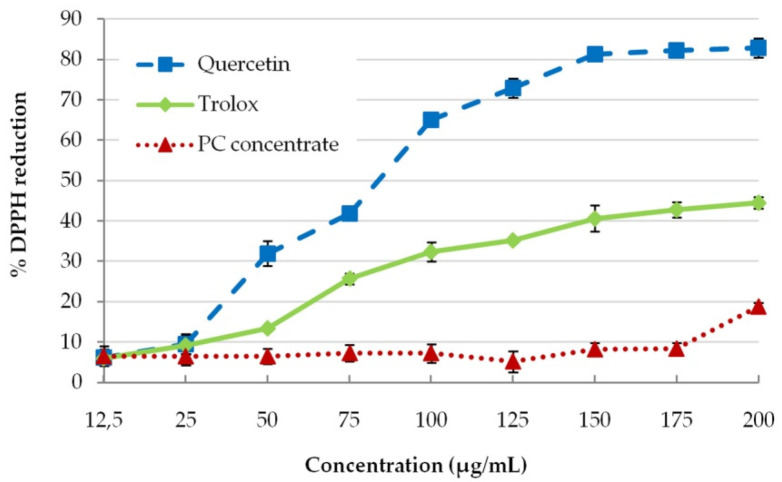
Percentage of reduced DPPH after adding of different concentrations of polyphenols from PC concentrate, trolox and quercetin.

A study of the antioxidant capacity of PC concentrate was provided in vitro. This study helped to evaluate the ability of PC concentrate to scavenge free radicals. The antiradical effect was studied using stable free radical DPPH. Trolox and quercetin were used as standard antioxidants (Figure 6, Table 3).

It was found that PC concentrate possessed lower DPPH radical scavenging activity than powerful antioxidants quercetin and trolox. We demonstrated that EC_50_ of PC concentrate was by 4.6 times higher than EC_50_ of quercetin and by 1.5 times higher than EC_50_ of trolox (Figure 6, Table 3).

#### 3.4.2. Liposome Oxidation Assay

**Figure 7:**
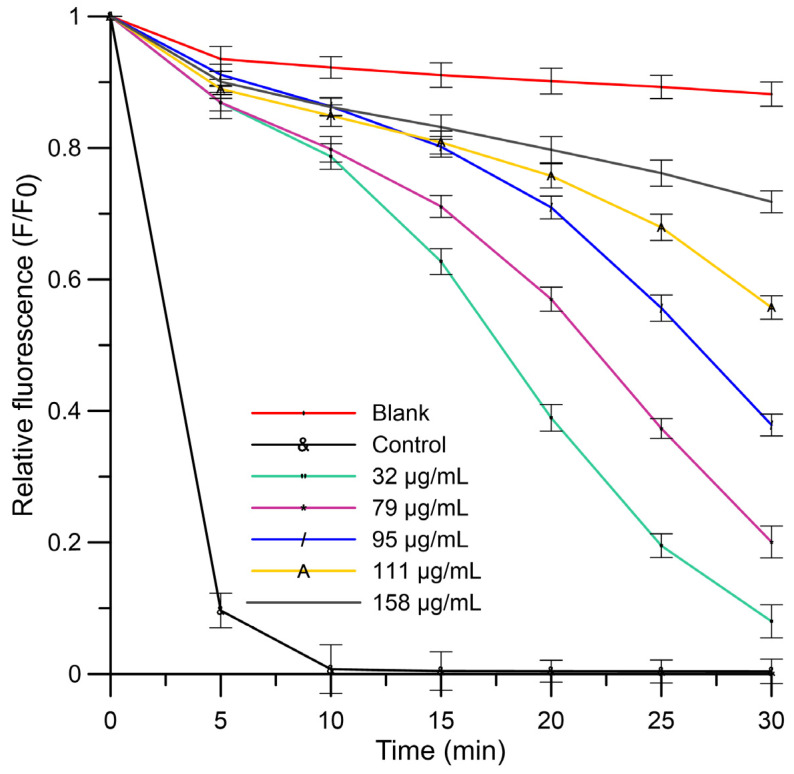
Relative fluorescence intensity of DPH-PA probe as a function of oxidation time of liposomes for AAPH radicals in the presence of PC concentrate at 32, 79, 95, 111 and 158 µg/mL concentrations. The relative change in fluorescence intensity F/F_0_ is a measure of the degree of lipid peroxidation (F_0_, fluorescence intensity in control sample; F, fluorescence intensity of samples in the presence of PC concentrate).

Using spectrophotometric method, the antioxidant activity was determined on the basis of the ability of PC concentrate to inhibit phosphatidylcholine lipids oxidation caused by alkyl radicals which were induced by the thermal decomposition of AAPH. The concentration of the extract which is responsible for 50% inhibition of the lipid peroxidation process IC_50_ (μg/mL) was taken as a measure of antioxidant activity. The results obtained are presented in Table 4. The relative fluorescence intensity kinetic curves of the probe DPH-PA in the presence and without PC concentrate and in relation to lipid membranes are shown in Figure 7.

Simultaneously with an increase in the concentration of PC concentrate, the fluorescence intensity increased in proportion to the inhibition of the lipid membrane oxidation process. Based on the plots of oxidation kinetics, the percentage of oxidation inhibition for extract after 30 min was calculated (Table 4). The kinetics of PC concentrate antioxidant effect may be described as follows: in the first phase of the reaction, namely, approximately the first 5 min of the reaction, the PC concentrate almost completely protects membrane lipids against AAPH-induced oxidation. Then, as the amount of antioxidants begins to deplete, a linear decrease in fluorescence intensity is observed. It is concluded that free radicals produced by AAPH in the aqueous environment are effectively eliminated by the components of the PC concentrate, which are located in the lipid membrane in their immediate vicinity.

#### 3.4.3. In Vivo Studies of PC Concentrate Antioxidant Effect

As a result of an imbalance between redox processes under the influence of external or internal factors in the body develops oxidative stress. Under these conditions oxidative modification of biomolecules, in particular lipids, proteins, and DNA, occurs [44]. Important biochemical markers of oxidative stress are the content of products of lipid peroxidation.

**Figure 8:**
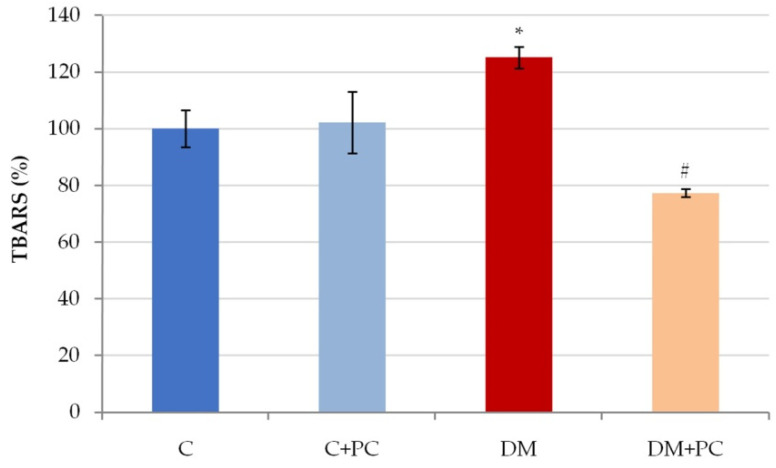
The content of TBARS in plasma of control rats, rats with experimental diabetes mellitus, with and without PC concentrate. Values of control rats are taken as 100%. C–normal untreated control animals; C + PC–animals that were per os administrated PC concentrate (daily dose of 45 mg/kg bw of polyphenolic complex for 14 days); DM–rats with experimental diabetes mellitus; DM + PC–animals with experimental diabetes mellitus that were per os administrated PC concentrate (daily dose of 45 mg/kg bw of polyphenolic complex for 14 days). *-significant difference compared to control (*p* < 0.05); #-significant difference compared to diabetes mellitus (*p* < 0.05).

It was found that the content of TBARS increased by 25% in plasma of diabetic rats when compared to control (Figure 8). In the case of PC concentrate influence, the level of TBARS decreased in plasma of diabetic rats by 48% compared to diabetes without PC concentrate consumption.

Aldehyde and ketone groups are formed in the side chain of amino acids when oxidative modification of blood plasma proteins occur. These groups react with 2,4-dinitrophenylhydrazine and form 2,4-dinitrophenylhydrazones, having a characteristic absorption spectrum. Aldehyde and keto derivatives of neutral character are registered at 370 nm, and of basic character–at 430 nm.

**Figure 9:**
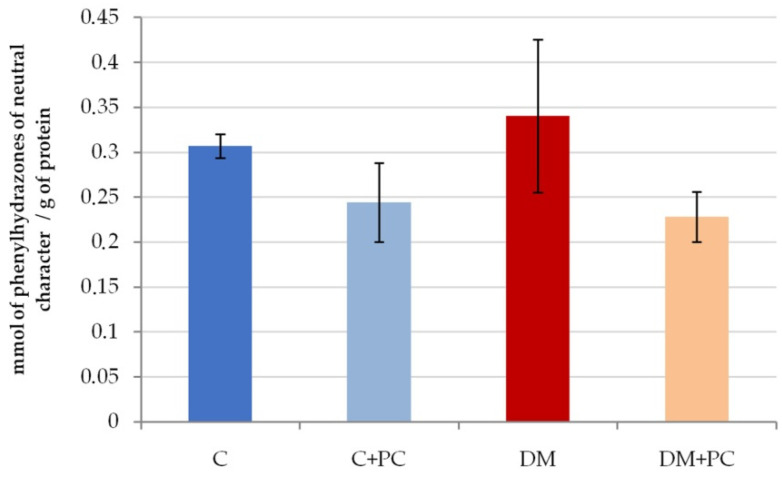
The content of proteins oxidative modification products of neutral character (370 nm) in plasma of control rats, rats with experimental diabetes mellitus, with and without PC concentrate. C–normal untreated control animals; C + PC–animals that were per os administrated PC concentrate (daily dose of 45 mg/kg bw of polyphenolic complex for 14 days); DM–rats with experimental diabetes mellitus; DM + PC–animals with experimental diabetes mellitus that were per os administrated PC concentrate (daily dose of 45 mg/kg bw of polyphenolic complex for 14 days).

**Figure 10:**
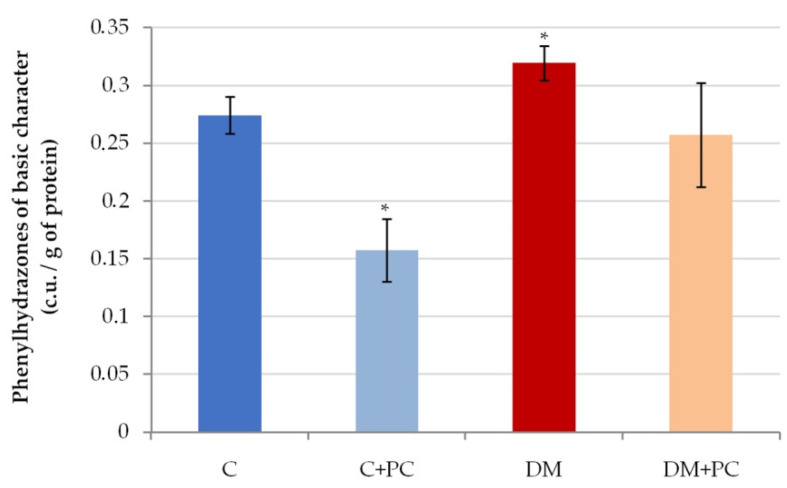
The content of proteins oxidative modification products of basic character (430 nm) in plasma of control rats, rats with experimental diabetes mellitus, with and without PC concentrate. C–normal untreated control animals; C + PC–animals that were per os administrated PC concentrate (daily dose of 45 mg/kg bw of polyphenolic complex for 14 days); DM–rats with experimental diabetes mellitus; DM + PC–animals with experimental diabetes mellitus that were per os administrated PC concentrate (daily dose of 45 mg/kg bw of polyphenolic complex for 14 days). *-significant difference compared to control (*p* < 0.05).

An increase of proteins oxidative modification products both neutral and basic character (by 1.13 and 1.16 times, respectively) was shown under conditions of diabetes mellitus compared to control (Figure 9 and Figure 10). The content of proteins oxidative modification products of neutral character decreased by 1.49 times in plasma of control and diabetes mellitus in the case of PC concentrate treatment (Figure 9) whilst the content of proteins oxidative modification products of basic character decreased by 1.24 times (Figure 10).

#### 3.4.4. The Activity of Antioxidant Enzymes of Plasma of Rats with Diabetes Mellitus under Red Wine Polyphenol Complex Concentrate Treatment

We studied the influence of PC concentrate on the activity of key antioxidant enzymes superoxide dismutase (SOD, EC 1.15.1.1), catalase (EC 1.11.1.6), and glutathione peroxidase (GPx, EC 1.11.1.9) in plasma of control and diabetic rats.

**Figure 11:**
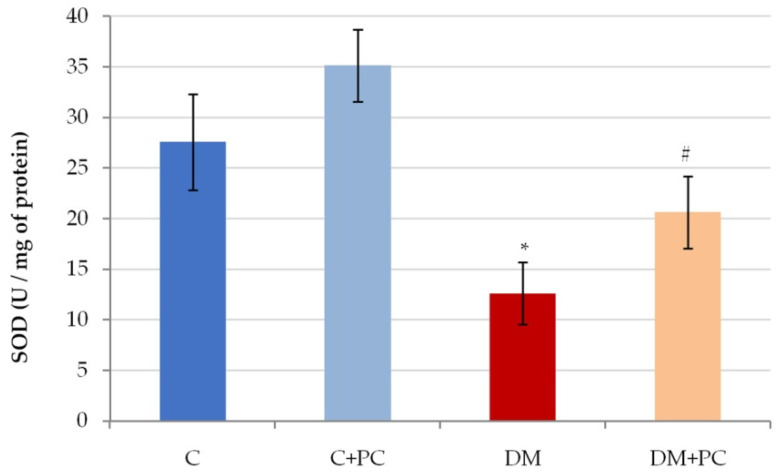
The activity of superoxide dismutase in plasma of control rats, rats with experimental diabetes mellitus, with and without PC concentrate. C–normal untreated control animals; C + PC–animals that were per os administrated PC concentrate (daily dose of 45 mg/kg bw of polyphenolic complex for 14 days); DM–rats with experimental diabetes mellitus; DM + PC–animals with experimental diabetes mellitus that were per os administrated PC concentrate (daily dose of 45 mg/kg bw of polyphenolic complex for 14 days). *-significant difference compared to control (*p* < 0.05); #-significant difference compared to diabetes mellitus (*p* < 0.05).

The activity of SOD was reduced by 2.5 times in plasma of rats with diabetes mellitus (Figure 11) compared to control. The effect may be caused by the inhibitory effect of Reactive Oxygen Species (ROS) on the enzyme. The administration of PC concentrate induced an increase of the enzyme activity in plasma of control animals by 1.13 times compared to control without treatment, as well as of diabetic animals by 1.64 times compared to diabetes without treatment. It should be mention that with the administration of PC concentrate to diabetic animals, the activity of SOD was still lower than the physiological level.

**Figure 12:**
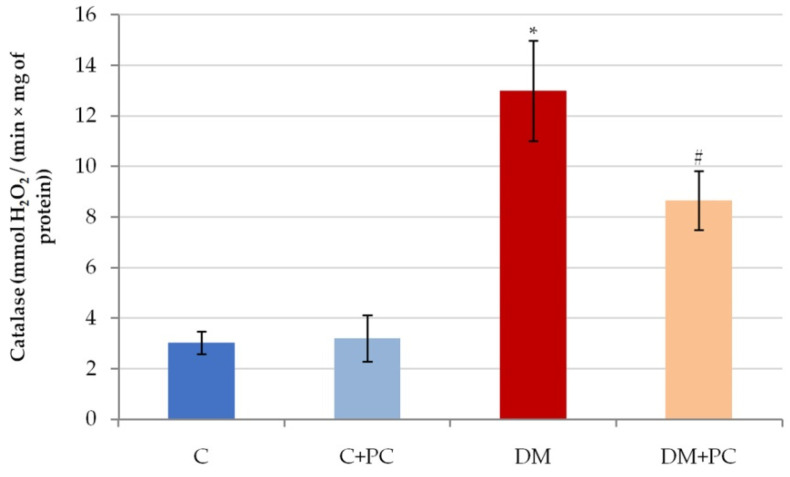
The activity of catalase in plasma of control rats, rats with experimental diabetes mellitus, with and without PC concentrate. C–normal untreated control animals; C + PC–animals that were per os administrated PC concentrate (daily dose of 45 mg/kg bw of polyphenolic complex for 14 days); DM–rats with experimental diabetes mellitus; DM + PC–animals with experimental diabetes mellitus that were per os administrated PC concentrate (daily dose of 45 mg/kg bw of polyphenolic complex for 14 days). *-significant difference compared to control (*p* < 0.05); #-significant difference compared to diabetes mellitus (*p* < 0.05).

The activity of catalase increased by 4.29 times in blood plasma under diabetes (Figure 12). PC concentrate administration induced a 1.5-fold decrease of catalase activity in plasma of diabetic rats. This may be caused by the ability of polyphenols to scavenge ROS, in particular Hydrogen peroxide, which is a substrate of catalase.

**Figure 13:**
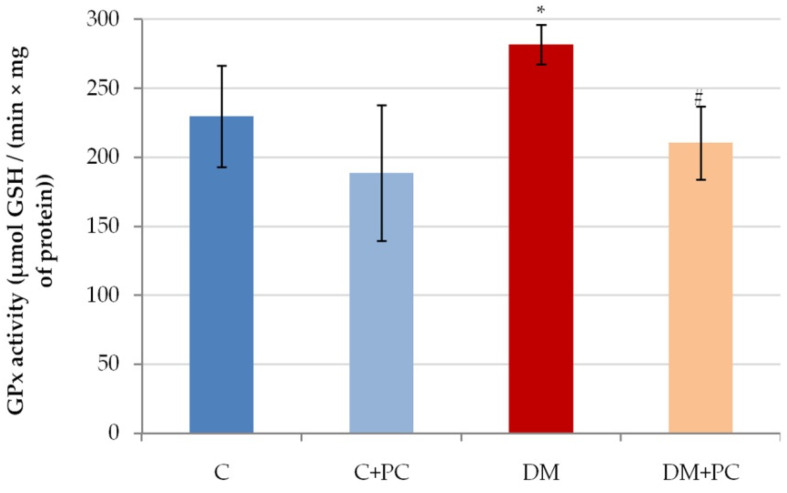
The activity of glutathione peroxidase in plasma of control rats, rats with experimental diabetes mellitus, with and without PC concentrate. C–normal untreated control animals; C + PC–animals that were per os administrated PC concentrate (daily dose of 45 mg/kg bw of polyphenolic complex for 14 days); DM–rats with experimental diabetes mellitus; DM + PC–animals with experimental diabetes mellitus that were per os administrated PC concentrate (daily dose of 45 mg/kg bw of polyphenolic complex for 14 days). *-significant difference compared to control (*p* < 0.05); #-significant difference compared to diabetes mellitus (*p* < 0.05).

We found out the increase of GPx activity by 1.23 times under experimental diabetes (Figure 13). PC concentrate administration induced a decrease in the enzyme activity in control animals by 1.22 times compared to control without PC concentrate administration. The index was lower by 1.34 times in plasma of diabetic animals in the case of PC concentrate administration when compared to diabetes mellitus.

## 4. Discussion

Diabetes has become one of the biggest health problems today. With the rapid growth of diabetes, many institutions and pharmaceutical companies have invested heavily in studying the pathogenesis and finding new treatments. Indeed, new drugs and technologies are constantly emerging. However, they do not help to control the rapid increase in the number of patients with diabetes. Along with that the more patients receive treatment, the more new cases are detected [45].

As a result, there is a problem of finding new drugs that will normalize blood glucose levels, correct diabetes-induced metabolic, morphological and functional disorders in cells and tissues, help to maintain redox balance, and prevent the development of oxidative stress. Such drugs can be produced on the basis of biologically active substances of natural origin. Such drugs are based on plant raw materials that have some of advantages, including the absence of side effects and the possibility of use not only for therapy but also for disease prevention.

The study aimed to assess the effect of red wine concentrate enriched with polyphenol compounds, on blood glucose levels, the activity of antioxidant enzymes in plasma of rats in experimental diabetes mellitus type 1. We also evaluated in vivo and in vitro antioxidant capacity of the PC concentrate. We hypothesized that the polyphenolic complex concentrate obtained from Ukrainian wine material will have hypoglycemic and antioxidant effects, as well as affect key hematological parameters in experimental type 1 diabetes mellitus due to the complex effect of biologically active substances. In addition, studied PC concentrate allows reducing the consumption of wine, in particular alcohol, but to ingest the required amount of phenolic compounds.

The oral glucose tolerance test is recommended by WHO and the American Diabetes Association as an important criterion for diagnostic of diabetes mellitus [46]. The glucose tolerance test allows getting information about the dynamics and degree of absorption of carbohydrates in peripheral tissues of the body and identifying possible violations of this process. We found an increase in fasting glucose in rats with experimental diabetes mellitus, along with the violation of cell tolerance to glucose and maintaining a high level of glycemia in 60 min of the post-absorption period. An unexpected result was that in the case of PC concentrate administration to animals with diabetes a glucose tolerance was partially recovered in the case of PC concentrate administration compared to diabetes, but not completely. Along with that polyphenols effectively reduced the level of glycated hemoglobin, which was elevated in diabetes melitus. Similar results were obtained by other scientists [16].

Polyphenols may affect blood glucose levels by various mechanisms. Hypoglycemic effect of polyphenols may be associated with inhibition of α-amylase and α-glucosidase and subsequent inhibition of carbohydrate digestion, slowing of intestinal glucose absorption, stimulation of insulin secretion, and protection of pancreatic β-cells against glucose toxicity. Polyphenols can inhibit the release of glucose by liver cells by affecting glucose metabolism in hepatocytes, as well as by activating insulin receptors and stimulating glucose uptake in both insulin sensitive and non-insulin sensitive tissues [18,20,47,48,49,50].

The natural polyphenolic complex reduces the erythrocyte membrane permeability to glucose and thus prevents glycated hemoglobin formation. Polyphenols incorporated into the plasma membrane or bind to its surface, protect it from attack by free radicals, inhibit oxidative chain reactions. In addition, polyphenols incorporation in the lipid bilayer causes a decrease in membrane fluidity and permeability due to the steric effect. This may cause inhibition of glucose diffusion across the erythrocyte membrane [51,52,53,54,55]. It has also been suggested that polyphenols inhibit GLUT1 glucose transporter, which may cause inhibition of glucose transport into erythrocytes [56].

The decrease of the indicator under PC concentrate administration to animals with diabetes mellitus indicates a stable long-term hypoglycemic effect of the studied concentrate, as glycated hemoglobin accumulates in the blood of patients with long-term diabetes mellitus.

Metformin (N,N dimethyl biguanidine) is commonly used for the treatment of diabetes mellitus type 2 [57,58,59]. A number of authors have shown that biologically active substances of natural origin have a less pronounced effect on blood glucose levels compared with metformin in rats with streptozotocin diabetes [60,61,62,63,64].

As a result of high glucose levels in the blood of patients with diabetes mellitus type 1 evolve a disturbance of kidneys filtration ability, decreased activity of enzymes of a renal epithelium which provide glucose resorption from primary urine, and glycosuria develops. A negative energy balance progresses. The cells try to adapt to this condition through more oxygen consumption [65]. Therefore, in the cells increases the probability of formation of aerobic metabolism byproducts–ROS [66,67] and oxidative stress develops. Oxidative stress is one of the determining factors in diabetes pathogenesis. Accordingly, complex drugs that inhibit oxidative stress development will be effective anti-diabetic drugs.

PC concentrate can reduce DPPH, but this ability was slightly lower than of the powerful antioxidants trolox and quercetin. This indicates a fairly high efficiency of polyphenols when used to overcome the effects of oxidative stress and increased levels of free radicals.

The previous research showed that the antioxidant effect of extracts rich in polyphenols is related to the ability of these components to eliminate free radicals that oxidize the membrane. The effectiveness of this process is related to the adsorption of antioxidant molecules to the polar region of the membrane, namely the place whose properties are modified by them. On the one hand, these molecules are able to increase the ordering of the surface of the model membrane as well as to limit the diffusion of free radicals into the membrane, on the other hand, they also are able to inactivate free radicals at the surface [68,69].

The results of the antioxidant studies were compared to a commonly used in food industry antioxidant, namely ascorbic acid, which was about 4.8 times more active than the PC concentrate. The obtained results, as well as the results of other researchers [70], suggest that the antioxidant activity of red wine concentrate may be largely explained due to its main phenolic components, i.e., anthocyanins and phenolic acids. To illustrate, our previous studies showed that anthocyanins such as cyanidin-3-*O*-galactoside (IC_50_ = 2.44 ± 0.55 µg/mL), cyanidin-3-*O*-rutinoside (IC_50_ = 3.99 ± 0.45 µg/mL) [71], as well as chlorogenic acid (IC_50_ = 2.45 ± 0.28 µg/mL) [46] are able to protect lipid membranes against peroxidation to a greater extent than the extract, i.e., a mixture of various compounds. This means that the antioxidant activity of the polyphenol mixture in the PC concentrate is lower than that of the isolated main components, a fact which may be explained as a result of mutual interactions. Numerous studies showed that polyphenols are these compounds, which are involved in redox reactions, so they can react as antioxidants or pro-oxidants depending on their concentration and environment [72,73]. In addition, different proportions within a mixture which contains single phenolic compounds give different results, ultimately showing a greater or lesser antioxidant activity. A study conducted by Samra, et al., 2011, [73] who tested the antioxidant activity of various polyphenolic compounds, showed that depending on the mixture and the relative concentration of each component, the observed effect was either antioxidant or pro-oxidative. The correlation between antioxidant activity and phenolic compounds was studied by numerous authors [74,75,76]. Wine consumption in in vivo studies in mice led to a significant reduction in TBARS and proteic carbonylation levels while compared to control groups. This demonstrates a protective effect of wine consumption on lipoperoxidation and proteic carbonylation. In addition, it was found that wine ingestion decreased significantly the activity of SOD, catalase, and GPx enzymes while compared to control groups [70]. In other studies, it was proved that Wistar rats with induced inflammation and oxidative stress were able to reduce malondialdehyde concentration in liver after receiving red wine doses [77].

Our results of the level of lipid peroxidation products and carbonyl groups of proteins in plasma of rats confirm oxidative stress development in diabetes mellitus type 1 conditions. The change in these biochemical parameters under PC concentrate administration indicates the antioxidant properties of polyphenols. It is known that polyphenols detoxify free radicals and in this way delay or inhibit the initiation step or interrupt the propagation step of lipid peroxidation [78]. Such effect of polyphenols has been proven by many scientists. It is known that polyphenols decreased lipid peroxidation and increased plasma total antioxidant capacity and even restored the depleted level of cellular antioxidant molecules in diabetic cells during oxidative stress [79,80,81,82]. Administration of metformin to rats with streptozotocin diabetes causes a decrease in the level of TBARS in the pancreas as well as biologically active substances of natural origin [60,63], but does not cause changes in the erythrocytes [83].

Redox balance is the basis of normal cell function. The increase in the content of prooxidants, including ROS, leads to oxidation processes activation and stable cytotoxic products accumulation, which are formed from almost all classes of biomolecules (lipids, proteins, nucleic acids, etc.). Normally, the accumulation of ROS and modified biomolecules stimulate gene expression or activation of enzymatic and non-enzymatic antioxidants. However, under pathological conditions, the intensity and degree of effectiveness of compensatory mechanisms, primarily enzymatic, change to varying degrees.

SOD is a unique family of metalloproteins whose physiological role is to take part in oxygen metabolism and to protect against direct and indirect damage by free radicals formed in oxygen conversion reactions [84,85,86,87,88]. We found a decrease in the activity of SOD in the plasma of rats with experimental diabetes mellitus. It is known, that persistent hyperglycemia in diabetes activates free radicals formation but also reduces the activity of antioxidant defense enzymes [89,90] due to oxidation by ROS or glycosylation of the active site of the enzyme [91].

Hydrogen peroxide formed in the SOD reaction further degrades under the action of catalase, peroxiredoxins, and GPx [88]. GPx affinity for low concentrations of hydrogen peroxide (1 μM) is higher than catalase affinity. Along with that GPx is able to convert only 8% of the total volume of hydrogen peroxide in the cell. This indicates the leading role of catalase in H_2_O_2_ destruction [92]. In addition to hydrogen peroxide conversion, GPx catalyzes the reduction of hydroperoxides of free and esterified fatty acids, hydroperoxides of phospholipids, nucleotides, nucleic acids, and possibly proteins into non-toxic molecules before they are converted to free radicals [93]. We found significantly increased activity of catalase and GPx in diabetes. PC concentrate determines the normalization of the activity of the studied antioxidant enzymes in plasma of rats with experimental diabetes. The obtained results indicate that the antioxidant properties of PC concentrate. This effect can be realized in two ways. Firstly, it may be due to the ability of polyphenols to scavenge an excessive amount of free radicals and prevent the inhibition of enzymes. Secondly, polyphenols activate the expression of antioxidant enzyme genes by binding to a specific enhancer (the antioxidant response element, ARE) [94]. Other authors established that antioxidant enzyme activity is not all regulated in the same manner. It was postulated, that modulation of CAT activity may be independent of gene expression in human erythrocytes in the case of wine consumption [95,96].

There is evidence in the literature that metformin as well as biologically active substances of natural origin reduces the activity of SOD in the pancreas of rats with streptozotocin diabetes. In addition, metformin does not affect the activity of catalase and glutathione peroxidase in the pancreas [60], but increases glutathione peroxidase activity in erythrocytes of rats with streptozotocin diabetes [83].

Our research showed that there was a tendency to decrease the number of erythrocytes in the peripheral blood and a significant decrease in the concentration of hemoglobin under conditions of diabetes mellitus. The obtained results are consistent with the data of other authors, although it is known that the concentration of total hemoglobin in diabetes can be both lower and higher than normal [97,98]. When PC concentrate was administrated to animals with diabetes, these figures increased. The probable reason for the normalization of total hemoglobin level in diabetes mellitus is the effect of polyphenol compounds on the processes of hemoglobin biosynthesis [94].

Although we did not confirm the effect of the obtained PC concentrate on tolerance to glucose, we proved its ability to adjust the level of glycated hemoglobin and influence key hematological parameters. In addition, the potent antioxidant activity of PC concentrate was confirmed in vivo, which is manifested in the ability to prevent protein oxidation, lipid peroxidation and correct antioxidant enzymes activity in plasma. This indicates the suppression of diabetic-induced oxidative stress by polyphenolic compounds from grape wine.

## 5. Conclusions

The red wine concentrate enriched with natural complex of polyphenols (PC concentrate) was obtained from dry red wine in the south of Ukraine. The composition of PC concentrate was analyzed. It was found that caftaric, coutaric and gallic acids among the phenolic acids were predominant components. The dominant anthocyanins were malvidin 3-*O*-glucoside and petunidin 3-*O*-glucoside.

It was revealed that polyphenolic compounds of PC concentrate modulate the level of hyperglycemia, normalize the number of erythrocytes and the concentration of hemoglobin in peripheral blood under conditions of experimental diabetes type 1. The antioxidant properties of PC concentrate in vitro and in vivo had also been proven.

PC concentrate enhances the antioxidant system and effectively prevents oxidative damage. PC concentrate treatment led to inhibition of lipid peroxidation and oxidative modification of proteins in plasma of rats with experimental diabetes mellitus. Studied PC concentrate caused the increase of the activity of SOD, and simultaneous reduce of the activity of catalase and GPx in plasma of diabetic rats. Additionally, this concentrate effectively inhibited peroxidation of phosphatidylocholine liposome under the stress condition.

Thereby, PC concentrate prevents the development of diabetes-induced oxidative stress in plasma of rats, which confirms the positive effect of these compounds in pathological conditions. Our results open up prospects for the usage of drugs, the main active ingredients of which are wine phenolic compounds, as adjuvants of complex therapy and prevention of complications of type 1 diabetes mellitus.

## Figures and Tables

**Table 1:** Physical and chemical properties of samples of dry wine made from Odessa Black grapes harvest in 2017.

Sample	The Volume Fraction of Ethyl Alcohol, %	Mass Content of Sugars, g/L	Mass Content of Titratable Acids, g/L	Mass Content of Total Extract Determination, g/L	The Efficiency of Phenolic Compounds Extraction, %	Mass Concentration of Dyes Compounds, mg/L	The Efficiency of Dyes Compounds Extraction, %
Dry red wine, variety Odessa Black 2017	14	1.6–2.0	4.8	25.0	83.0	1543.0	89.0

**Table 2:** Identification and the content (mg/100 mL) of anthocyanins and phenolic acids of red wine (1) and PC concentrate (2) by UPLC-ESI-qTOF-MS/MS) and HPLC-PDA.

Peak. No.	*t_R_* _(HPLC)_ (min)	Compound	UV λ_max_ (nm)	[M − H]^−^/[M + H]^+^ (*m/z*)	Other Ions (*m/z*)	Content (mg per 100 mL)	
						1	2
**Anthocyanins [M + H]^+^**							
1	6.70	Dpglc	522	465.1026	303.0530	5.99 ± 0.16	6.58 ± 0.13
2	8.05	Cy glc	514	449.1092	287.0562	1.35 ± 0.12	1.66 ± 0.20
3	8.94	Pt glc	523	479.1210	317.0676	6.80 ± 0.06	6.99 ± 0.20
4	10.35	Pnglc	516	463.1239	301.0727	13.92 ± 0.15	14.91 ± 0.47
5	11.02	Mv glc	526	493.1339	331.0825	39.28 ± 0.30	42.18 ± 1.97
6	11.45	Vit A-Pnglc	503	531.1199	369.0592	2.13 ± 0.13	1.93 ± 0.57
7	12.08	Dp acetyl glc	510	507.1139	303.0494	6.64 ± 0.05	6.98 ± 0.40
8	12.08	Vit A- Mv glc		561.1240	399.0722		
9	13.03	Vit A-Mv acetyl glc	507	603.1283	399.0681	Tr	Tr
10	13.27	Cy acetyl glc	512	491.1145	287.0562	1.09 ± 0.01	1.23 ± 0.07
11	14.23	Pt acetyl glc	527	521.1305	317.0676	1.67 ± 0.52	1.50 ± 0.56
12	15.85	Pn acetyl glc	516	505.1350	301.0727	3.09 ± 0.06	3.60 ± 0.29
13	16.30	Mv acetyl glc	529	535.1441	331.0825	8.41 ± 0.19	8.99 ± 0.85
14	17.53	Vit A-Pn*p*-coumglc	508	677.1500	369.0592	0.44 ± 0.14	0.41 ± 0.02
15	17.96	Vit A-Mv *p*-coumglc	512	707.1570	399.0722	0.39 ± 0.17	0.34 ± 0.02
16	18.33	Cy *p*-coumglc	506	595.2069	287.0562	0.23 ± 0.22	0.19 ± 0.01
17	18.82	Pt *p*-coumglc	527	625.1439	317.0676	0.10 ± 0.02	0.12 ± 0.06
18	19.57	Pn*p*-coumglc	522	609.1618	301.0727	1.73 ± 0.07	1.97 ± 0.03
19	19.80	Mv *p*-coumglc	528	639.1744	331.0788	3.66 ± 0.04	4.00 ± 0.15
20	21.23	Mv 3-glc-4-vin ph adduct	502	609.1618	447.1086	0.52 ± 0.00	0.55 ± 0.01
		**Total**				**97.44**	**104.13**
**Phenolic acid [M − H]^−^**							
21	1.54	Gallic acid	272	169.0116		29.10 ± 1.90	34.90 ± 1.77
22	2.76	Protocatechuic acid	260/295	153.0164		3.99 ± 0.18	4.91 ± 0.48
23	3.91	Caftaric acid	326	311.0404	179.0342	66.85 ± 3.83	69.37 ± 1.71
24	5.86	Coutaric acid	312	295.0457	163.0379	41.97 ± 2.15	44.12 ± 2.46
		**Total**				**141.91**	**153.30**

Tr, trace; Cy, cyanidin; Dp, delphinidin; Pt, petunidin; Pn, peonidin; Mv, malvidin; glc, glucose; acetyl glc, (6-*O*-acetyl)-glucoside; p-coumglc, (6-*O*-*p*-coumaroyl)-glucoside; vit A, vitisin A; vin ph, vinyl phenol.

**Table 3:** Half maximal effective concentration (EC_50_) for PC concentrate, trolox and quercetin for DPPH radical scavenging activity.

Compound	EC_50_ (µg/mL)
PC concentrate	357.59 ± 75.23
Trolox	229.83 ± 6.12
Quercetin	77.78 ± 1.51

**Table 4:** Antioxidant activity parameters (IC_50_) for PC concentrate into liposomes membrane. The oxidation was induced by AAPH.

Compound	IC_50_ (µg/mL)
PC concentrate	108.38 ± 11.19
L(+) ascorbic acid	22.80 ± 2.19 *

* the result is taken from [43] with the kind permission of the authors.

## Data Availability

Data is contained within the article.
